# Utilization and Perceptions of Hydroxyurea Therapy Among Adult Patients With Sickle Cell Disease in Al Ahsa, Saudi Arabia: A Cross-Sectional Study

**DOI:** 10.7759/cureus.64666

**Published:** 2024-07-16

**Authors:** Intithar H Alherz, Zainab J Al-Nass, Mohammed A Alkadi

**Affiliations:** 1 Preventive Medicine, Al-Ahsa Health Cluster, Al-Hofuf, SAU; 2 Preventive Medicine, Qatif Central Hospital, Qatif, SAU; 3 Internal Medicine, Al-Ahsa Health Cluster, Al-Hofuf, SAU

**Keywords:** saudi arabia, clinical outcomes, barriers, patient perceptions, utilization patterns, hydroxyurea therapy, sickle cell disease

## Abstract

Background: Sickle cell disease (SCD) is a prevalent genetic disorder characterized by abnormal hemoglobin formation, resulting in severe complications. Hydroxyurea (HU) therapy has demonstrated efficacy in reducing SCD-related complications; however, its utilization patterns and patient perceptions remain underexplored, particularly in the Al Ahsa region of Saudi Arabia.

Objective: This cross-sectional study aimed to assess the prevalence of HU usage among adult patients with SCD in Al Ahsa; identify the barriers to starting, maintaining, and discontinuing HU therapy; and evaluate the patient-reported outcomes associated with its use.

Methods: Data were collected through face-to-face surveys and medical record reviews of adult SCD patients attending outpatient clinics in the Hereditary Blood Diseases Center of Al Ahsa, Saudi Arabia, between December 2023 and March 2024. Descriptive statistics and inferential analyses were performed using SPSS version 26.

Results: A total of 345 adult SCD patients were included, with a mean age of 34.12 ± 11.1 years. Most participants were male (58.6%) and unmarried (55.4%). HU utilization was reported by 57.1% of the participants, with the highest adherence observed among older age groups (p = 0.001). Significant improvements in pain severity, hospitalization rates, and quality of life were reported among HU users (p < 0.001). Common barriers to HU use included concerns about side effects, lack of medical justification, and absence of medical advice.

Conclusion: This study provides valuable insights into the utilization and perceptions of HU therapy among adults with SCD in Al Ahsa, Saudi Arabia. Addressing identified barriers and promoting patient education are crucial for optimizing therapy adherence and improving clinical outcomes in this population.

## Introduction

Sickle cell anemia (SCA) is a common genetic condition inherited as an autosomal recessive disorder [1]. It primarily affects the development of red blood cells, resulting in the formation of hemoglobin S (Hb S), an abnormal type of hemoglobin that causes red blood cells to become sickle-shaped and inflexible. These abnormal cells clump together in the arteries, blocking blood flow to various organs and causing various life-threatening complications [2].

Globally, approximately 7% of the world's population are carriers of hemoglobinopathies. Sickle cell disease (SCD) affects millions of people worldwide, with a particularly high prevalence among populations from sub-Saharan Africa, Spanish-speaking regions in the Western Hemisphere (South America, Cuba, and Central America), Saudi Arabia, India, and Mediterranean countries [3].

In Saudi Arabia, SCD is highly prevalent, particularly in the southern and eastern regions of the country. The first reported case was documented in 1960, notably in the eastern province. The prevalence in this region is markedly higher at 145 cases per 10,000 population compared to the southern region (24 cases/10,000 population), western region (12 cases/10,000 population), and central region (six cases/10,000 population). The prevalence of SCD trait carriers in Saudi Arabia ranges from approximately 2% to 27%. The Saudi Premarital Screening Program estimates that 0.26% of the adult population are trait carriers, while 4.2% are diagnosed with the disease [4].

From a management perspective, acute pain related to SCD in both adults and children is commonly addressed using a short course (five to seven days) of nonsteroidal anti-inflammatory drugs in addition to opioids. However, to prevent and reduce the frequency of painful crises, hydroxyurea therapy is considered a viable option. Hydroxyurea increases the synthesis of fetal hemoglobin in most patients with SCA, exerting only mild myelotoxicity. By inhibiting sickling, elevated levels of fetal hemoglobin may decrease the frequency of painful crises [5].

The Multicenter Study of Hydroxyurea (MSH) in SCA demonstrated the potential of hydroxyurea therapy to ameliorate the clinical course of SCA in some adults experiencing three or more painful crises per year [6]. Additional benefits include reductions in the frequency of chest syndrome and the need for transfusions. Consequently, hydroxyurea received approval from the Food and Drug Administration (FDA) for the treatment of adults with HbSS and frequent episodes (three or more annually) of severe pain in 1998. Furthermore, indications for hydroxyurea use extend to acute chest syndrome within the last 12 months or symptomatic anemia [7,8].

Study objective

This study aims to investigate the actual usage of hydroxyurea among adult SCD patients in Al Ahsa, Saudi Arabia. It seeks to determine the prevalence of hydroxyurea utilization; identify any underuse by healthcare providers; understand patient-reported barriers to initiating, adhering to, and interrupting hydroxyurea therapy; and propose recommendations to enhance its utilization. These insights are crucial for improving preventive measures to manage the sickle cell crisis effectively in the Al Ahsa community.

## Materials and methods

Study design

A cross-sectional study, approved by King Fahad Hospital-Hofuf's Institutional Review Board Committee in the Al Ahsa Health Cluster, facilitated data collection at a single point in time, providing a snapshot of the population.

Study location and population

The study was conducted in Al Ahsa City, Saudi Arabia, among adult patients with SCD attending outpatient clinics of the hereditary blood disease center.

Inclusion Criteria

Participants were patients with SCD receiving regular outpatient follow-up at the Hereditary Blood Disease Center, aged 18 years or older.

Exclusion Criteria

Patients with contraindications to hydroxyurea use were excluded, including those with hypersensitivity to hydroxyurea or its components, and those with severe bone marrow depression, including leukopenia, thrombocytopenia, or severe anemia [9].

Data collection tool and technique

Face-to-face surveys and medical record reviews were employed to collect data on demographic information, SCD status, and hydroxyurea use. Data collection occurred at the outpatient clinics of the Hereditary Blood Disease Center in Al Ahsa City between December 2023 and March 2024.

The data were collected, reviewed, and then fed to the Statistical Package for Social Sciences version 26 (IBM Inc., Armonk, NY, USA). All statistical methods used were two-tailed with an alpha level of 0.05 considering significance if the P value is less than or equal to 0.05. Descriptive analysis was done by prescribing frequency distribution and percentage for study variables including SCD cases personal data, SCD phenotypes, HU use, and changes after use. SCD-related complications and reasons for not using HU were graphed. Cross tabulation for showing factors associated with HU use, the relation between SCD complications and the prevalence of using HU, and the relation between commitment to HU use and the improvement in SCD complications. The significance of the relation was assessed using the Pearson chi-square test.

## Results

A total of 399 cases were identified for the study: 54 cases were excluded as their age was above 16 but less than 18 years, and notably, eligible 345 cases with SCD were included. The patients’ ages ranged from 18 to 64 years, with a mean age of 34.12 ± 11.1 years old. The main bulk (138, 30.0%) of the age category was <30 years. Exactly 201 (58.6%) cases were males, 191 (55.4%) were married, and 141 (40.9%) were single. Many of the participants were unemployed (83, 24.1%), followed by students (73, 21.3%). The uttermost participants had monthly incomes of less than 5000 SR (211, 61.2%), and 103 (29.9%) had monthly incomes of 5000-10000 SR. The lion’s share (172, 49.9%) had a university level of education or above, and 117 (33.9%) had a secondary level of education. Considering the genotype of SCD, it was Hb SS among 131 (38.0%) and Hb S beta-thalassemia among 85 (24.6%). Hereditary persistence of fetal hemoglobin HbF was observed in 45 (13.0%), while 84 (24.4%) had unknown genotypes due to missed data or unreported interpretation. Only 75 (21.7%) cases had other comorbidities, mainly diabetes mellitus (DM) and hypertension (HTN) (Table 1).

**Table 1:** Bio-demographic characteristics of the study patients with sickle cell disease in Al Ahsa City (n = 345). The data are represented as (N and %) .

Bio-demographic data	n	%
Age in years		
<30	138	30.0%
30-39	84	24.3%
40+	123	35.7%
Gender		
Male	201	58.3%
Female	144	41.7%
Marital status		
Single	141	40.9%
Married	191	55.4%
Divorced	9	2.6%
Widow	4	1.2%
Job title		
Unemployed	83	24.1%
Student	74	21.4%
Governmental employee	59	17.1%
Private employee	60	17.4%
Housewife	54	15.7%
Retired	9	2.6%
Freelancing	6	1.7%
Monthly income		
<5000 SR	211	61.2%
5000-10000 SR	103	29.9%
11000-20000 SR	24	7.0%
>20000 SR	7	2.0%
Educational level		
Below secondary	56	16.2%
Secondary	117	33.9%
University/above	172	49.9%
Phenotype of SCD		
Hb SS	131	38.0%
Hb S beta-thalassemia	85	24.6%
Hereditary persistence of fetal hemoglobin HbF	45	13.0%
I don't know	84	24.4%
Other comorbidities		
Yes	75	21.7%
No	270	78.3%

Spectrum of sickle cell complications among the participants

**Figure 1:**
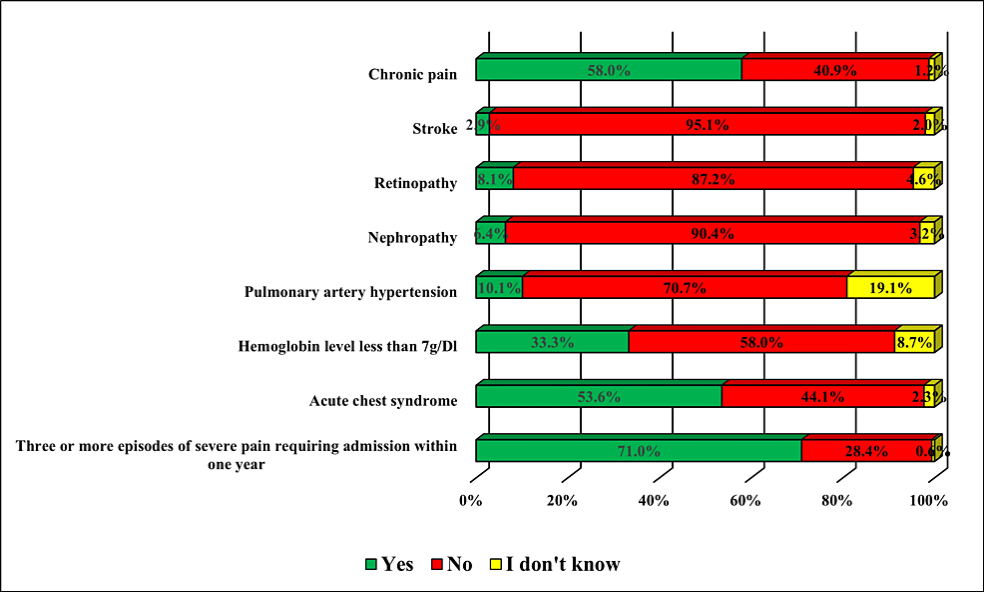
Complications of sickle cell disease among the study cases in Al Ahsa, Saudi Arabia. The data are represented as (%).

With reference to SCD complications among the study cases, the most reported were having three or more episodes of severe pain requiring hospitalization within one year (71.0%), chronic pain (58.0%), acute chest syndrome (53.6%), chronic anemia manifested by hemoglobin level less than 7 g/dL (33.3%), and pulmonary artery hypertension (10.1%). The least reported complications included stroke (2.9%) and retinopathy (8.1%) (Figure 1).

Prevalence and reasons for hydroxyurea use among SCD patients

About 197 (57.1%) of the study's SCD cases frequently used HU, while seven (2%) infrequently used HU and were not regular users. On the other hand, 108 (31.3%) did not use and 33 (9.7%) quit using the drug (Table 2).

**Table 2:** Prevalence of hydroxyurea use among sickle cell disease patients in Al Ahsa City. The data are represented as (N and %).

HU use	n	%
Do you use hydroxyurea?		
I use.	197	57.1%
I’m not a regular user.	7	2.0%
No	108	31.3%
I quit using.	33	9.7%
Reasons for using hydroxyurea		
I had three or more episodes of severe pain requiring admission within one year.	192	55.7%
I was suffering from hemolysis and chronic anemia.	80	23.2%
I was suffering from acute chest syndrome.	134	38.8%
I was suffering from chronic pain.	144	41.7%
I have used it since childhood.	7	2.0%
Pulmonary artery hypertension.	25	7.2%
I don’t know.	1	0.3%
I had priapism.	11	3.2%
I was suffering from retinopathy.	7	2.0%
I was suffering from nephropathy.	17	4.9%
I had a stroke.	6	1.7%
After using HU reported improvements		
Less severe pain attacks	162	47.0%
Fewer hospitalizations	160	46.4%
My quality of life has improved.	175	50.7%
My chronic pain has improved.	121	35.1%
Less blood transfusions	54	15.7%
I never had acute chest syndrome again.	89	25.8%
Symptoms of pulmonary artery hypertension improved	15	4.3%
Nephropathy improved (or did not get worse)	11	3.2%
Fewer priapism attacks	6	1.7%

The most reported reasons for using HU were experiencing three or more episodes of severe pain requiring admission within one year in 192 (75.9%) cases or suffering from chronic pain in 144 (41.7%) cases or acute chest pain in 134 (38.8%). This was followed by those who were suffering from hemolysis and chronic anemia (80, 23.2%) (Table 2).

The use of hydroxyurea resulted in reported improvements primarily in terms of enhancing the quality of life in 175 cases (50.7%) of SCD and less severe pain attacks in 162 (47.0%) cases, followed by fewer hospitalizations in 160 (46.4%) cases. To a lesser extent, 121 (35.1%) cases reported improvement in chronic pain, and 89 (25.8%) never had acute chest syndrome again (Table 2).

Among 33 participants who have interrupted use of HU, the most reported reasons included side effects of treatment in 29 (8.4% of the total cases) and pregnancy in four (1.2% of the total cases) (Table 3).

**Table 3:** Reasons for interrupting using hydroxyurea among the study cases with sickle cell disease. The data are represented as (N and %).

	Yes	No	Not applicable
	n (%)	n (%)	n (%)
Unavailability of treatment	0 (0.0%)	17 (4.9%)	328 (95.1%)
Pregnancy (for females)	4 (1.2%)	6 (1.7%)	335 (97.1%)
Use of stem cells	0 (0.0%)	17 (4.9%)	328 (95.1%)
Side effects of treatment	29 (8.4%)	2 (0.6%)	314 (91.0%)

The current work revealed that there was high significance (p = 0.001) of its use and the age group of the participants. About 50 (59.5%) HU users were aged between 30 and 39 years old, and 72 (58.5%) HU users were around 40. The number of male HU users was higher (124, 61.7%), compared to the female HU users (73, 50.7%). Widowed cases were four (100%) and those who had high monthly income were seven (100%) (Table 4).

**Table 4:** Factors associated with hydroxyurea use among the study cases of SCD. The data are represented as (N and %).

Variables	Use of hydroxyurea				p-value
	Yes	No	I’m not a regular user.	I quit using.	
	n (%)	n (%)	n (%)	n (%)	
Age in years					0.001*
<30	75 (54.3%)	58 (42.0%	1 (0.7%)	4 (2.9%)	
30-39	50 (59.5%)	19 (22.6%)	2 (2.4%)	13 (15.5%)	
40+	72 (58.5%)	31 (25.2%)	6 (4.9%)	14 (11.4%)	
Gender					0.227
Male	124 (61.7%)	55 (27.4%)	5 (2.5%)	17 (8.5%)	
Female	73 (50.7%)	53 (36.8%)	4 (2.8%)	14 (9.7%)	
Marital status					0.042*
Single	79 (56.0%)	54 (38.3%)	4 (2.8%)	4 (2.8%)	
Married	108 (56.5%)	52 (27.2%)	5 (2.6%)	26 (13.6%)	
Divorced	6 (66.7%)	2 (22.2%)	0 (0.0%)	1 (11.1%)	
Widow	4 (100.0%)	0 (0.0%)	0 (0.0%)	0 (0.0%)	
Job title					0.452
Unemployed	44 (53.0%)	24 (28.9%)	3 (3.6%)	12 (14.5%)	
Student	42 (56.8%)	30 (40.5%)	1 (1.4%)	1 (1.4%)	
Governmental employee	37 (62.7%)	16 (27.1%)	1 (1.7%)	5 (8.5%)	
Private employee	36 (60.0%)	19 (31.7%)	1 (1.7%)	4 (6.7%)	
Housewife	28 (51.9%)	16 (29.6%)	3 (5.6%)	7 (13.0%)	
Retired	7 (77.8%)	1 (11.1%)	0 (0.0%)	1 (11.1%)	
Freelancing	3 (50.0%)	2 (33.3%)	0 (0.0%)	1 (16.7%)	
Monthly income					0.094
<5000 SR	116 (55.0%)	65 (30.8%)	8 (3.8%)	22 (10.4%)	
5000-10000 SR	55 (53.4%)	39 (37.9%)	1 (1.0%)	8 (7.8%)	
11000-20000 SR	19 (79.2%)	4 (16.7%)	0 (0.0%)	1 (4.2%)	
>20000 SR	7 (100.0%)	0 (0.0%)	0 (0.0%)	0 (0.0%)	
Educational level					0.202
Below secondary	28 (50.0%)	20 (35.7%)	3 (5.4%)	5 (8.9%)	
Secondary	67 (57.3%)	31 (26.5%)	4 (3.4%)	15 (12.8%)	
University/above	102 (59.3%)	57 (33.1%)	2 (1.2%)	11 (6.4%)	
Type of SCD					0.699
Hb SS	74 (56.5%)	43 (32.8%)	4 (3.1%)	10 (7.6%)	
Hb S beta-thalassemia	54 (63.5%)	20 (23.5%)	2 (2.4%)	9 (10.6%)	
Hereditary persistence of fetal hemoglobin HbF	27 (60.0%)	15 (33.3%)	0 (0.0%)	3 (6.7%)	
I don't know	42 (50.0%)	30 (35.7%)	3 (3.6%)	9 (10.7%)	
Other comorbidities					0.587
Yes	40 (53.3%)	23 (30.7%)	3 (4.0%)	9 (12.0%)	
No	157 (58.1%)	85 (31.5%)	6 (2.2%)	22 (8.1%)	

A total of 158 (64.5%) cases with three or more episodes of severe pain requiring hospitalization within one year used HU with high significance (p < 0.001). Moreover, 113 (61.1%) cases with acute chest syndrome used HU compared to others without (p = 0.548). Moreover, cases with stroke (5, 59%) and retinopathy (18, 68.2%) were HU users with high significance (p = 0.001, 0.002). Other complications of SCD were not significantly associated with using HU (Table 5).

**Table 5:** Relationship between SCD complications and the prevalence of using hydroxyurea. The data are represented as (N and %).

Complications	Use of hydroxyurea				p-value
	Yes	No	I’m not a regular user.	I quit using.	
	n (%)	n (%)	n (%)	n (%)	
Three or more episodes of severe pain requiring hospitalization within one year					0.000*
Yes	158 (64.5%)	54 (22.0%)	7 (2.9%)	26 (10.6%)	
No	38 (38.8%)	53 (54.1%)	2 (2.0%)	5 (5.1%)	
I’m not sure	1 (50.0%)	1 (50.0%)	0 (0.0%)	0 (0.0%)	
Acute chest syndrome					0.548
Yes	113 (61.1%)	50 (27.0%)	4 (2.2%)	18 (9.7%)	
No	79 (52.0%)	56 (36.8%)	5 (3.3%)	12 (7.9%)	
I’m not sure	5 (62.5%)	2 (25.0%)	0 (0.0%)	1 (12.5%)	
Hemolysis or chronic anemia					0.738
Yes	64 (55.7%)	35 (30.4%)	5 (4.3%)	11 (9.6%)	
No	117 (58.5%)	63 (31.5%)	4 (2.0%)	16 (8.0%)	
I’m not sure	16 (53.3%)	10 (33.3%)	0 (0.0%)	4 (13.3%)	
Pulmonary artery hypertension					0.371
Yes	24 (68.6%)	11 (31.4%)	0 (0.0%)	0 (0.0%)	
No	136 (55.7%)	78 (32.0%)	6 (2.5%)	24 (9.8%)	
I’m not sure	37 (56.1%)	19 (28.8%)	3 (4.5%)	7 (10.6%)	
Priapism					0.055
Yes	7 (53.8%)	2 (15.4%)	0 (0.0%)	4 (30.8%)	
No	99 (64.3%)	41 (26.6%)	5 (3.2%)	9 (5.8%)	
I’m not sure	91 (51.2%)	65 (36.5%)	4 (2.1%)	18 (10.1%)	
Stroke					0.001*
Yes	5 (50.0%)	4 (40.0%)	1 (10.0%)	0 (0.0%)	
No	190 (57.9%)	103 (31.4%)	8 (2.4%)	27 (8.2%)	
I’m not sure	2 (28.6%)	1 (14.3%)	0 (0.0%)	4 (57.1%)	
Nephropathy					0.021
Yes	15 (68.2%)	5 (22.7%)	1 (4.5%)	1 (4.5%)	
No	179 (57.4%)	100 (32.1%)	7 (2.2%)	26 (8.3%)	
I’m not sure	3 (27.3%)	3 (27.3%)	1 (9.1%)	4 (36.4%)	
Retinopathy					0.002*
Yes	18 (64.3%)	6 (21.4%)	0 (0.0%)	4 (14.3%)	
No	174 (57.8%)	97 (32.2%)	9 (3.0%)	21 (7.0%)	
I’m not sure	5 (31.3%)	5 (31.3%)	0 (0.0%)	6 (37.5%)	
Chronic pain					
Yes	119 (59.5%)	55 (27.5%)	6 (3.0%)	20 (10.0%)	
No	78 (55.3%)	51 (36.2%)	3 (2.1%)	9 (6.4%)	0.028
I’m not sure	0 (0.0%)	2 (50.0%)	0 (0.0%)	2 (50.0%)	

The current study revealed a significant relationship between adherence to HU and improvement of most SCD complications. Less severe pain attacks were recorded by 143 (88.3%) of the adherent HU users and 19 (11.7%) of the non-adherent HU users with high significance (p ≤ 0.001). The same scenario was encountered regarding fewer hospitalizations, acute chest syndrome, less blood transfusion, chronic pain improvement, and improved quality of life in 141 (88.1%), 81 (91.0%), 46 (85.2%), 105 (86.8%), and 157 (89.7%) of the adherent HU users. Moreover, all cases of improved pulmonary artery hypertension (15, 100%) were adherent to use HU. The reverse was revealed for priapism. The non-adherent HU users (four, 66.7%) improved more than the adherent HU users (two, 33.3%) (Table 6).

**Table 6:** Relationship between adherence to hydroxyurea and the improvement in sickle cell disease (SCD) complications. The data are represented as (N and %).

Improvement of SCD complications	I am committed to using hydroxyurea.		I’m not committed to using hydroxyurea.		p-value
	n	%	n	%	
Less severe pain attacks	143	88.3%	19	11.7%	0.000*
Fewer hospitalizations	141	88.1%	19	11.9%	0.000*
I never had acute chest syndrome again.	81	91.0%	8	9.0%	0.000*
Less blood transfusions	46	85.2%	8	14.8%	0.000*
Symptoms of pulmonary artery hypertension improved	15	100.0%	0	0.0%	0.004*
Fewer priapism attacks	2	33.3%	4	66.7%	0.000*
My chronic pain has improved.	105	86.8%	16	13.2%	0.000*
Nephropathy improves (or does not get worse)	10	99.9%	1	9.1%	0.111
My quality of life has improved.	157	89.7%	18	10.3%	0.000*

A total of 66 (47.5%) cases did not use HU as they received medical advice, and there is no medical justification for its use; about 37 (26.6%) did not receive any medical advice for its use. Fear of side effects was also reported, mainly fertility effect (49, 35.3%), pregnancy worry (45, 32.4%), hair loss (53, 38.1%), immunity suppression (48, 34.5%), decreased platelets (35, 25.2%), and skin ulcers (32, 23.0%) (Table 7).

**Table 7:** Reasons for not using hydroxyurea among study cases with sickle cell disease (n = 139)*. The data are represented as (N and %). *Total cases: non-users and cases with interruptions

Reasons	Yes		No				Not sure		
	n	%		n	%	n		%	
Received medical advice and there is no medical justification for its use	66	47.5%		59	42.4%	14		10.1%	
I have not received any medical advice for its use.	37	26.6%		94	67.6%	8		5.8%	
I am concerned about possible side effects.									
Fertility	49	35.3%		29	20.9%	61		43.9%	
Pregnancy	45	32.4%		43	30.9%	51		36.7%	
Hair loss	53	38.1%		61	43.9%	25		18.0%	
Immune suppression	48	34.5%		68	48.9%	23		16.5%	
Low platelets	35	25.2%		72	51.8%	32		23.0%	
Skin ulcers	32	23.0%		75	54.0%	32		23.0%	

## Discussion

As the accurate number of users of HU among adult patients with SCD in Al Ahsa is not well-documented, this study provides valuable insights into its actual utilization. By examining real-world data, it seeks to identify any underutilization of HU among practitioners and explore potential barriers to its initiation, adherence, and interruption as reported by patients.

The current study revealed that most of the study participants were in their middle age and were males. Approximately one-third of the participants had the Hb SS subtype, while about one-fourth had Hb S beta-thalassemia, with fewer participants having hereditary persistence of fetal hemoglobin (HbF). Notably, the percentage of fetal Hb is considered high in the Al Ahsa population [10]. Interestingly, a study conducted in Saudi Arabia, which explored Saudi HbS homozygotes with the Arab Indian haplotype, found that the mean HbF level was 19.2 ± 7.0% [11]. These findings could potentially explain the overall lower mortality rate in adults affected by the high percentage of fetal Hb compared to other populations with lower fetal Hb levels [12].

The pain in adults with SCD is the rule rather than the exception, which is revealed by several observational studies [13,14]. The current study also revealed that the most reported complications with SCD were acute painful crisis followed by chronic pain, acute chest syndrome, and chronic anemia with hemoglobin levels less than 7 g/dL, but less frequent complications such as pulmonary artery hypertension, retinopathy, and nephropathy with priapism were reported.

In terms of identifying HU users, the current study found that 57.1% of the individuals with SCD reported using HU, with a high level of adherence among the majority of users, evidenced by a non-adherence rate of only 2%.

A separate study conducted in the Eastern Province of Saudi Arabia in 2022 reported that 38% of SCD patients were using HU at that time. This variance from our study's finding may be attributed to differences in sample size and the inclusion of a mixed population comprising both pediatric and adult patients [15]. Furthermore, in our study, there is a direct relationship between age and the use of HU, as most users are 30 years of age or more. This is consistent with a retrospective study reviewed at Ann & Robert H. Lurie Children’s Hospital of Chicago between June 2012 and June 2017, which demonstrated that young adults with SCD had significantly higher HU adherence compared to children and adolescents [16].

There are factors that could support increasing adherence with age, including throughout adolescence. The transition to adulthood allows for even greater independence, control, and self-care, which can be motivating to patients [16].

In a study by Madkhali et al. in Saudi Arabia, Jazan, it was reported that 57.6% of SCD patients were using HU, a rate comparable to our findings. However, notably, the study also noted that 81.6% of HU users reported low adherence [17]. Similarly, another cross-sectional study conducted in the US revealed almost the same percentage, with 56.3% of patients on HU, and an adherence rate reaching up to 80.6% [18].

However, a higher prevalence was noted in Riyadh. Azmet et al. reported that nearly 78% of patients in a different study conducted in Riyadh, Saudi Arabia, between May 2017 and January 2018, were using HU [19]. Moreover, in Jeddah, 61% of SCD patients were on HU in 2021 [20]. Notably, both studies primarily involved pediatric populations.

In contrast to previous studies conducted in Europe between 2008 and 2017, our current study reveals a notably higher percentage of HU usage among patients. Specifically, our findings exceed the reported percentages of HU utilization in those studies, which ranged from 25% to 40% [21-23]. In addition, a study by Jose et al. in Oman reported that approximately 43% of SCD patients were receiving HU treatment [24].

Several studies provide insight into HU adherence among SCD patients. A study using Texas Medicaid data revealed that only 35% of SCD patients were adherent to HU [25]. Similarly, another retrospective study utilizing Texas Medicaid data found that 20.9% were adherent to HU, indicating suboptimal adherence among most patients. In addition, a cross-sectional study involving adolescents and young adults with SCD showed that only 35% of participants were adherent to HU [26]. These findings highlight the challenges associated with achieving optimal adherence to HU therapy among individuals with SCD.

In our study, the most commonly reported reasons for using HU were painful crises, hemolysis, chronic anemia, and acute chest syndrome. These reasons align with the approved indications for HU use based on the MSH study [6,7].

According to our current analysis, it is challenging to identify a significant relationship between the off-label use of HU to manage other complications of SCD and any observable improvements. This lack of association could be attributed to the low percentage of users among off-label indications. Despite indirect evidence suggesting the potential benefits of HU in modifying the long-term sequelae of several SCD complications, particularly priapism [27], pulmonary hypertension [28], and nephropathy [29]. These findings are primarily based on observational studies and clinical trials are still required for definitive evidence.

Importantly, the impact of HU use among study participants demonstrated several positive outcomes, including reduced severity of pain attacks, decreased hospitalizations, enhanced quality of life, alleviation of chronic pain, and diminished need for blood transfusions. This is consistent with other studies.

A multi-center cross-sectional study in Nigeria found that among SCD patients using HU, 92.5% had fewer pain crises, 84.8% had fewer transfusion needs, and 84.6% had fewer hospital admissions [30].

Moreover, a meta-analysis found that HU significantly lowered transcranial Doppler velocity, tricuspid regurgitant velocity, and reduced the risk of albuminuria, potentially reducing organ damage [31]. These findings collectively highlight the beneficial effects of HU therapy in improving various aspects of disease management and reducing the risk of complications in individuals with SCD.

Chronic pain is a significant concern among adults with SCD, constituting one of the most frequently reported complications in our study, affecting up to 58% of individuals. Similarly, findings from a daily diary study by Smith et al. in 2008 revealed that 54% of adults with SCD experience pain 51% of the time, with 29% experiencing pain almost daily [13]. In another observational study, the majority of participants reported experiencing chronic pain for three to six days each week, totaling 84.1% [32].

Our study highlights the potential benefit of HU in managing chronic pain through off-label use, given the absence of optimal short-term or long-term modifying therapeutic agents for chronic pain control in SCD patients [5]. While our results suggest a positive effect of HU on chronic pain management, definitive conclusions should be drawn from clinical trials with predefined inclusion criteria for chronic pain.

The most commonly reported reasons for not using HU in our study included the lack of medical justification for its use, absence of medical advice, and concerns about potential side effects, particularly related to fertility, pregnancy, hair loss, and immunity suppression.

Similarly, in another cross-sectional study addressing barriers to HU use, it was found that worry about side effects was a common barrier for both participants on and not on HU, with percentages of 16.8% and 19.8%, respectively [18]. However, another study that focused on unintentional barriers found that participants more frequently cited factors falling into the unintentional nonadherence category (70%), such as forgetfulness and external influences [33]. These findings underscore the diverse array of challenges hindering HU use and adherence among individuals with SCD.

Continuing counseling every sickle cell patient about the potential benefits of HU during routine clinical surveillance, while also addressing any concerns regarding potential side effects, could prove highly beneficial. This proactive approach has the potential to boost HU utilization rates and mitigate barriers to its adoption. In addition, prioritizing research to establish the safety of HU use among pregnant patients, based on the latest available studies is crucial for ensuring comprehensive care and informed decision-making [34].

Limitations of the study

The study's limitations include potential interviewer and recall biases due to reliance on face-to-face survey responses. In addition, accurate diagnosis of certain complications of SCD may have been challenging, and objective tools for measuring improvement were lacking, relying instead on patient-reported outcomes. Furthermore, the absence of a standardized scale or protocol for assessing quality of life improvement after HU exposure is notable. Lastly, the study's regional focus on the Al Ahsa region may limit the generalizability of findings to other regions, as patient characteristics may vary.

## Conclusions

In conclusion, this study provides valuable insights into the utilization and perceptions of HU therapy among adults with SCD in Al Ahsa, Saudi Arabia. By identifying barriers to treatment adherence, such as concerns about side effects and lack of medical advice, healthcare providers can tailor interventions to address these challenges and optimize clinical outcomes.

Patient education plays a pivotal role in empowering individuals with SCD to actively engage in their care and make informed decisions about their treatment. By promoting education and overcoming barriers to HU utilization, healthcare professionals can enhance disease management and improve the overall well-being of patients in Al Ahsa.
